# Comment from the Enhancing Burn Rehabilitation Special Edition Editors on “Fractional CO_2_ Laser for Pediatric Hypertrophic Scars: Lessons Learned from a Prematurely Terminated Split-Scar Trial”

**DOI:** 10.3390/ebj6010016

**Published:** 2025-03-13

**Authors:** Dale W. Edgar, Colleen M. Ryan, Marianne K. Nieuwenhuis, Ulrike Van Daele, Jill M. Cancio

**Affiliations:** 1 Burn Injury Research Node, Institute for Health Research, The University of Notre Dame Australia, Fremantle, WA 6160, Australia; 2 Fiona Wood Foundation, Fiona Stanley Hospital, Murdoch, WA 6150, Australia; 3 Massachusetts General Hospital, Harvard Medical School, Shriners Hospitals for Children, Boston, MA 02114, USA; cryan@mgh.harvard.edu; 4 Alliance of Dutch Burn Care, Burn Centre Groningen, Martini Hospital, 9700 RM Groningen, The Netherlands; nieuwemk@mzh.nl; 5 Department of Human Movement Sciences, University of Groningen, University Medical Center Groningen, 9713 AV Groningen, The Netherlands; 6 Research Group on Healthy Ageing, Allied Health Care and Nursing, Hanze University of Applied Sciences, 9747 AS Groningen, The Netherlands; 7 Research Group MOVANT (Movement Antwerp), Department of Rehabilitation Sciences and Physiotherapy, University of Antwerp, 2000 Antwerp, Belgium; ulrike.vandaele@uantwerpen.be; 8 OSCARE, Organisation for Burns, Scar Aftercare and Research, 2170 Antwerp, Belgium; 9 U.S. Army Burn Center, U.S. Army Institute of Surgical Research, Ft. Sam Houston, San Antonio, TX 78234, USA; jill.m.cancio.civ@health.mil

The Editors thank the investigators for displaying tenacity, evidenced by the significant revisions that were necessary to complete this project. Their published study describes a randomized CO_2_ ablative laser treatment protocol, applied to pediatric burn scars [1]. It highlights challenges and provides readers with key learnings about the design and conduct of intervention trials during the post-burn rehabilitation phase, especially when investigating scar treatments for pediatric burn patients. This trial was diligently designed as a randomized, controlled, clinical intervention trial. However, the original trial was abandoned due to the negative impact of the COVID-19 pandemic on the ability to recruit ambulatory patients and carers, who were ultimately seeking an intervention to improve the symptoms and quality of their mature scars. It is worth reiterating that the investigators also note that motivation to join this study was likely impacted by the choice to apply CO_2_ ablative laser treatment under general anesthetic and to only half of the studied scar surface area.

After the pilot study, the investigators proceeded with the trial as a combined feasibility and pilot trial [2]. As this was not the initial intention, readers must consider the contextual factors in their interpretation of the presented results, because traditional feasibility and/or pilot trial assessments of the investigative process were applied ad hoc in this study [3,4]. The interpretation of the clinical results in the report must be approached with extreme caution and require further confirmation due to the heterogeneous, small sample including only female participants, spanning multiple developmental age stages and with scars at vastly different points of maturation. Based on this, specific caution must be applied in the interpretation of cluster analysis, again with respect to recruited numbers and repeated (scar) assessments, which were not available for all participants.
